# Coupled environmental and demographic fluctuations shape the evolution of cooperative antimicrobial resistance

**DOI:** 10.1098/rsif.2023.0393

**Published:** 2023-11-01

**Authors:** Lluís Hernández-Navarro, Matthew Asker, Alastair M. Rucklidge, Mauro Mobilia

**Affiliations:** Department of Applied Mathematics, School of Mathematics, University of Leeds, Leeds LS2 9JT, UK

**Keywords:** eco-evolutionary dynamics, antimicrobial resistance, cooperation, environmental variability, coexistence, fluctuations

## Abstract

There is a pressing need to better understand how microbial populations respond to antimicrobial drugs, and to find mechanisms to possibly eradicate antimicrobial-resistant cells. The inactivation of antimicrobials by resistant microbes can often be viewed as a cooperative behaviour leading to the coexistence of resistant and sensitive cells in large populations and static environments. This picture is, however, greatly altered by the fluctuations arising in volatile environments, in which microbial communities commonly evolve. Here, we study the eco-evolutionary dynamics of a population consisting of an antimicrobial-resistant strain and microbes sensitive to antimicrobial drugs in a time-fluctuating environment, modelled by a carrying capacity randomly switching between states of abundance and scarcity. We assume that antimicrobial resistance (AMR) is a shared public good when the number of resistant cells exceeds a certain threshold. Eco-evolutionary dynamics is thus characterised by demographic noise (birth and death events) coupled to environmental fluctuations which can cause population bottlenecks. By combining analytical and computational means, we determine the environmental conditions for the long-lived coexistence and fixation of both strains, and characterise a *fluctuation-driven* AMR eradication mechanism, where resistant microbes experience bottlenecks leading to extinction. We also discuss the possible applications of our findings to laboratory-controlled experiments.

## 1. Introduction

Environmental conditions, such as temperature, pH or available resources, endlessly change over time and shape the fate of natural populations. For instance, microorganisms often live in volatile environments where resource abundance fluctuates between mild and harsh conditions, and regimes of feast alternate with periods of famine [1–3]. How environmental variability (EV), generally referring to changes not caused by the organisms themselves (e.g. supply of abiotic resources), affects species diversity is a subject of intense debate and research (e.g. [4–13]). Demographic noise (DN) arising from randomness in birth and death events in finite populations is another source of fluctuations. DN is negligible in large populations and strong in small ones, where it can lead to species fixation, when one species takes over the population, or to extinction, and hence can permanently set the make-up of a community [14–17]. The dynamics of the population composition (evolutionary dynamics) is often coupled with that of its size (ecological dynamics) [18], resulting in its *eco-evolutionary dynamics* [19–21].

When EV influences the size of a population, it also modulates the DN strength, leading to a coupling of DN and EV [21–26]. This interdependence is potentially of great relevance to understand eco-evolutionary dynamics of microbial communities. The coupling of DN and EV can lead to population bottlenecks, where new colonies consisting of few individuals are prone to fluctuations [27–30], and plays an important role in the eco-evolutionary dynamics of antimicrobial resistance (AMR) [31,32].

The rise of AMR is a global threat responsible for millions of deaths [33]. Understanding how AMR evolves and what mechanisms can possibly eradicate the resistance to antimicrobials are therefore questions of great societal relevance and major scientific challenges. A common mechanism of AMR involves the production by resistant cells, at a metabolic cost, of an extra or intracellular enzyme inactivating antimicrobial drugs [34–36]. When the number of resistant cells exceeds a certain threshold, there are enough drug-inactivating enzymes, and the protection against antimicrobial drugs is shared with sensitive cells that can thus also resist antimicrobial drugs at no metabolic cost. However, below the resistant population threshold, only resistant microbes are protected against the drug (enzyme availability is limited and it can only inactivate the drug in the vicinity of resistant cells). AMR can hence be viewed as a thresholded cooperative behaviour where widespread antimicrobial inactivation is a form of public good. This results in the spread of resistant microbes below the threshold, while sensitive cells thrive under high enzymatic concentration (above threshold). Hence, in static environments and large populations, both sensitive and resistant strains survive antimicrobial treatment and coexist in the long run [36–39]. In this work, we show that this picture can be greatly altered by the joint effect of demographic and environmental fluctuations, often overlooked, but ubiquitous in microbial communities that commonly evolve in volatile environments, where they can be subject to extreme and sudden changes [27–31,40,41].

Motivated by the problem of the evolution of AMR, here we study the coupled influence of EV and DN on the eco-evolutionary dynamics of a population of two species, one antimicrobial-resistant strain and the other sensitive to antimicrobials. In our model, we assume that AMR is a cooperative behaviour above a certain threshold for the number of resistant microbes, and the microbial community is subject to environmental fluctuations that can cause population bottlenecks. Here, EV involves random switches of the carrying capacity, causing the population size to fluctuate, while the antimicrobial input is kept constant. We thus study how the joint effect of EV and DN affects the fixation and coexistence properties of both strains, determining under which environmental conditions either of them prevail or if they both coexist for extended periods. This allows us to identify and fully characterise a *fluctuation-driven* AMR eradication mechanism, where environmental fluctuations generate transients that greatly reduce the resistant population and DN can then lead to the extinction of AMR.

In the next section, we introduce the model and discuss our methods. We present our results in §3, where we first describe the main properties of the (*in silico*) model evolving under a fluctuating environment, and then study its properties analytically. In §§3.1–3.3, we analyse the population dynamics in the large population limit, and then the model’s fixation properties in static environments. In §3.4, we characterise the fixation and coexistence of the strains in fluctuating environments, and discuss in detail the fluctuation-driven eradication of AMR arising in the regime of intermediate switching. Section 4 is dedicated to the discussion of the influence of EV on the strains fraction and abundance (§4.1), and to a review of our modelling assumptions (§4.2). Our conclusions are presented in §5. Technical and computational details are given in the electronic supplementary material [42].

## 2. Methods and models

Microbial communities generally evolve in volatile environments: they are subject to suddenly changing conditions [40,41], and fluctuations can play an important role in their evolution [4,5,7,8,10–12,31]. For instance, fluctuating nutrients may be responsible for population bottlenecks leading to feedback loops and cooperative behaviour [27–30], while sensitivity to antimicrobials depends on cell density and its fluctuations [36–39,43]. Here, we study the eco-evolutionary dynamics of cooperative AMR by investigating how a well-mixed microbial community evolves under the continued application of a drug that hinders microbial growth when the community is subject to fluctuating environments. The evolutionary dynamics of the microbial community is modelled as a multivariate birth-and-death process [14,44,45], whereas to model the fluctuating environment we assume that the population is subject to a time-varying binary carrying capacity [23,24,46–48].

### 2.1. Microbial model

We consider well-mixed co-cultures composed of an antimicrobial-resistant cooperative strain (denoted by *R*) and a defector type sensitive to antimicrobials (labelled *S*), under a constant input of antimicrobial drug, inspired by a chemostat laboratory set-up. The population, of total size *N*, hence consists of *N*_*R*_ resistant and *N*_*S*_ sensitive microbes, with *N* = *N*_*R*_ + *N*_*S*_. Note that, since we later introduce EV as switches in the carrying capacity, the total population will fluctuate accordingly. A frequent mechanism of AMR relies on the production of an *enzyme hydrolysing the antimicrobial drug* in their surroundings [34–36]. Here, we assume that each *R* cell produces the enzyme at a constant rate, regardless of the antimicrobial concentration, which is inspired by typical lab experiments, e.g. with resistance gene-bearing plasmids [36,37]. When the number of *R* is high enough, the overall concentration of resistance enzyme in the medium suffices to inactivate the drug for the entire community: the enzyme hydrolyses the drug and sets it below the *minimum inhibitory concentration* (MIC), therefore acting as a public good and protecting *S* as well. This mechanism can hence lead to AMR as a cooperative behaviour [34–36], for instance, by means of the *β*-lactamase resistance enzyme for the general *β*-lactam family of antibiotics [49] (see §4.2 for non-shared resistance mechanisms).

Here, we model this AMR mechanism by assuming that *R* acts as a cooperative strain when the number of *R* cells (proxy for resistance enzyme concentration) exceeds a fixed threshold *N*_th_, i.e. *R* cells are cooperators when *N*_*R*_ ≥ *N*_th_, while they retain for themselves the benefit of producing the protecting enzyme when *N*_*R*_ < *N*_th_ [36–39,43]. The effective regulation of public good production by means of a population threshold has been found in a number of microbial systems (e.g. [27,39,50–52]), and is consistent with a slower microbial growth cycle with respect to the fast time scale of enzyme production and dispersion. In this work, we study the AMR evolution as a form of cooperative behaviour under demographic and environmental fluctuations. Assuming fixed-volume fluctuating environments, the threshold for AMR cooperation is here set in terms of *R* abundance (rather than its concentration), see §4.

**Figure 1.**
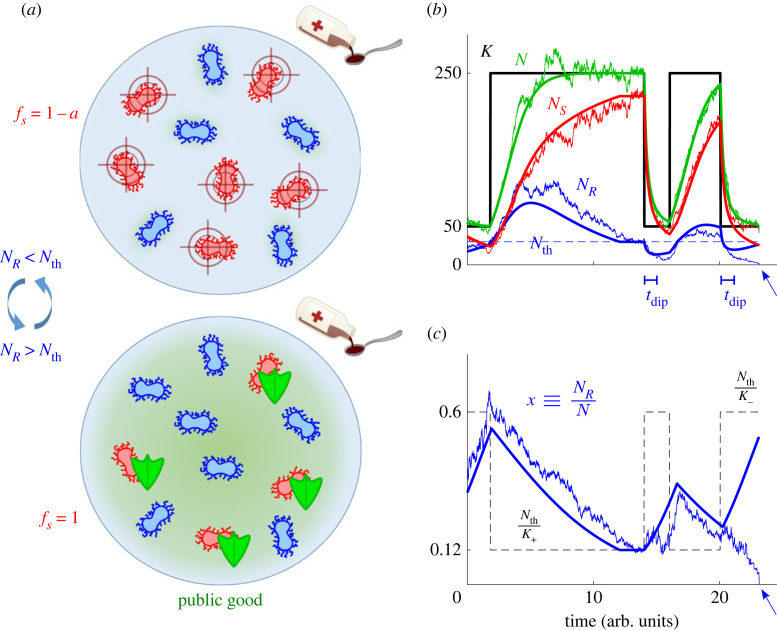
Microbial community model. (*a*) Top: when the abundance of *R* (blue microbes) is below the cooperation threshold *N*_th_, antimicrobial drug hinders the growth rate of *S* (red microbes) and *R* cells have a growth advantage. Bottom: AMR becomes cooperative when the number of *R* exceeds *N*_th_ and these generate enough *resistance enzyme* (public good in green shade) to hydrolyse the antimicrobial drug below the MIC for the whole medium, so that protection against the drug is shared with *S* (with green shields). (*b*) Temporal eco-evolution dynamics of the microbial community for example parameters *s* = 0.2, *a* = 0.5, *K*_−_ = 50, *K*_+_ = 250, *ν* = 0.2 and *δ* = 0.6; thick black line shows the sample path of the time-switching carrying capacity *K*(*t*), with a cooperation threshold *N*_th_ = 30 (blue line); thick solid lines depict the *N* → ∞ piecewise deterministic (deterministic between two switches of *K*) process defined by equations (3.2) and (3.3) for the total microbial population (*N*, green), number of *R* (*N*_*R*_ = *Nx*, blue) and number of *S* (*N*_*S*_ = *N*(1 − *x*), red); noisy lines show an example stochastic realisation of the full model under the joint effect of demographic and environmental fluctuations. In the absence of DN, *R* can experience bumps and dips (thick blue line), and *t*_dip_ indicates the mean time to reach the bottom of a dip from its inception; see §3.4. In the presence of DN, fluctuations about the dip can lead to the extinction of *R* (blue arrow). (*c*) *R* fraction *x* = *N*_*R*_/*N* for the same sample path of varying environment as in (*b*); line styles as in (*b*); the dashed black line shows the stable *R* fraction in each environment as *K*(*t*), driven by *ξ*(*t*), switches in time.

In our model, *R* microbes have a constant birth rate independent of the biostatic drug hindering microbial growth [53–55],^1^ with fitness *f*_*R*_ = 1 − *s*, where 0 < *s* < 1 captures the extra metabolic cost of constantly generating the resistance enzyme. The birth rate of *S* depends on the public good abundance: when *N*_*R*_ < *N*_th_, the enzyme concentration is low (below cooperation threshold) and the antimicrobial drug is above the MIC, the *S* fitness *f*_*S*_ is thus lower than *f*_*R*_, with *f*_*S*_ = 1 − *a*, where 1 > *a* > *s* and *a* encodes growth rate reduction caused by the drug. When *N*_*R*_ ≥ *N*_th_, the *R* abundance is above the cooperation threshold. This triggers the AMR cooperative mechanism: the drug is inactivated (below MIC), and the *S* birth rate, with *f*_*S*_ = 1, is then higher than that of *R*, see figure 1*a*. Denoting by *x* ≡ *N*_*R*_/*N* the fraction of *R* in the population, here *S* fitness is

$$f_{S}=1-a\theta \;[N_{\mathrm{th}}-N_{R}]=1-a\theta \;[x_{\mathrm{th}}(N)-x],$$

where *θ*[*z*] is the Heaviside step function, defined as *θ*[*z*] = 1 if (*z* > 0) and *θ*[*z*] = 0 otherwise, and *x*_th_(*N*) ≡ *N*_th_/*N* is the fraction of *R* at the cooperation threshold. The average population fitness is $\bar{f}=f_{R}N_{R}/N+f_{S}N_{S}/N$. In this setting, this population evolves according to the multivariate birth–death process [16,44,45] defined by the reactions

2.1 $$\begin{array}{cc} & N_{R/S}\overset{T_{R/S}^{+}}{\to }N_{R/S}+1\\ \mathrm{and}\;\;\; & N_{R/S}\overset{T_{R/S}^{-}}{\to }N_{R/S}-1, \end{array}$$

occurring with transition rates [21–23,25]

2.2 $$\begin{array}{cc} & T_{R}^{+}=\frac{\;f_{R}}{\bar{f}}N_{R}=\frac{(1-s)\;N_{R}}{1-a\theta [N_{\mathrm{th}}-N_{R}]+(a\theta [N_{\mathrm{th}}-N_{R}]-s)N_{R}/N},\;T_{R}^{-}=\frac{N}{K}N_{R}\\ \mathrm{and}\; & T_{S}^{+}=\frac{\;f_{S}}{\bar{f}}N_{S}=\frac{(1-a\theta [N_{\mathrm{th}}-N_{R}])\;N_{S}}{1-a\theta [N_{\mathrm{th}}-N_{R}]+(a\theta [N_{\mathrm{th}}-N_{R}]-s)N_{R}/N},\;T_{S}^{-}=\frac{N}{K}N_{S}, \end{array}$$

with growth limited by the logistic death rate *N*/*K* (so that the total population *N* follows the standard logistic dynamics in the mean field limit; see equation (3.1)), where *K* is the carrying capacity that is here assumed to be a time-fluctuating quantity; see below. Moreover, we have normalised *f*_*R*/*S*_ by the average fitness $\bar{f}$ for mathematical convenience, without loss of generality (see §4.2). This corresponds to the growth rate of each strain to be given by its fitness relative to the average population’s fitness, a common assumption in the context of biological and evolutionary processes [6,16,56], which allows us to establish a neat relationship between our multivariate birth–death process and the classical Moran process. The latter is the reference birth–death-like process used to model the evolution of idealised populations of constant total size [14,16,57–59]. The link with the Moran process enables us to take advantage of its well-known properties, in particular the exact results for the fixation probability and mean fixation time [14,16,57,59], to characterise analytically many features of our eco-evolutionary model (see §§3.2 and 3.3, and electronic supplementary material, section D [42]).

### 2.2. Environmental fluctuations and master equation

In addition to demographic fluctuations stemming from random birth and death events (see equation (2.1)), we model EV as sudden changes in the available resources, such as in cycles of feast and famine [1–3,60]. We implement this by letting the carrying capacity be a binary time-fluctuating random variable *K*(*t*) ∈ {*K*_−_, *K*_+_}, with *K*_+_ > *K*_−_, as broadly used in eco-evolutionary modelling [22–26,32,46–48,61,62]. This allows us to simply model sudden extreme changes in the population size, particularly the formation of population bottlenecks [25,27–30,63], providing us with a theoretical counterpart of commonly used laboratory experimental chemostat set-ups [63–66]; see §4.2.

For simplicity, we consider that *K*(*t*) is driven by the coloured dichotomous Markov noise (DMN) *ξ*(*t*) = { − 1, 1} that randomly switches between *K*_−_ and *K*_+_. The DMN is an important example of bounded noise, with finite correlation time that is easy to simulate accurately (see electronic supplementary material, section A [42]) and amenable to analytical progress, and hence often employed in modelling evolutionary processes in fluctuating environments [21–23,25,26,46–48,67]. The dynamics of the DMN is defined by the simple reaction [46,47,67]

2.3 ξ⟶−ξ,

endlessly occurring at rate (1 − *δξ*)*ν*, where −1 < *δ* < 1. Here, we always consider the DMN at stationarity where *ξ* = ±1 with probability (1 ± *δ*)/2. The stationary DMN ensemble average is thus 〈*ξ*(*t*)〉 ≡ ((1 + *δ*)/2) − ((1 − *δ*)/2) = *δ* and its auto-covariance (auto-correlation up to a constant) is 〈*ξ*(*t*)*ξ*(*t*′)〉 − 〈*ξ*(*t*)〉〈*ξ*(*t*′)〉 = (1 − *δ*^2^) e^−2*ν*|*t*−*t*′|^, where *ν* is both half the inverse of the correlation time and average switching rate. We thus consider that the binary switching carrying capacity is [21,23,25,26]

2.4 $$K(t)=\frac{1}{2}[K_{+}+K_{-}+\xi (t)(K_{+}-K_{-})],$$

and *K*(*t*) thus switches from a state where resources are abundant (*K*_+_) to another state where they are scarce (*K*_−_) with rates *ν*_+_ ≡ *ν*(1 − *δ*) and *ν*_−_ ≡ *ν*(1 + *δ*) according to

$$K_{-}\overset{\nu_{-}}{\underset{\nu_{+}}{⇌}}K_{+}.$$

Environmental statistics can be characterised by the mean switching rate *ν* ≡ (*ν*_−_ + *ν*_+_)/2 and by *δ* ≡ (*ν*_−_ − *ν*_+_)/(*ν*_−_ + *ν*_+_) that encodes the environmental switching bias: when *δ* > 0, on average, more time is spent in the environmental state *ξ* = 1 than *ξ* = −1, and thus *K* = *K*_+_ is more likely to occur than *K* = *K*_−_ (symmetric switching arises when *δ* = 0). The time-fluctuating carrying capacity (2.4) modelling *environmental fluctuations* is responsible for the time-variation of the population size, and is coupled with the birth-and-death process (2.1) and (2.2).

The master equation (ME) giving the probability *P*(*N*_*R*_, *N*_*S*_, *ξ*, *t*) for the population to consist of *N*_*R*_ and *N*_*S*_ cells in the environmental state *ξ* at time *t* is [44]

2.5 $$\begin{array}{rl} & \frac{\partial P(N_{R},N_{S},\xi ,t)}{\partial t}\\ & =(E_{R}^{-}-1)[T_{R}^{+}P(N_{R},N_{S},\xi ,t)]+(E_{S}^{-}-1)[T_{S}^{+}P(N_{R},N_{S},\xi ,t)]\\ & \;+(E_{R}^{+}-1)[T_{R}^{-}P(N_{R},N_{S},\xi ,t)]+(E_{S}^{+}-1)[T_{S}^{-}P(N_{R},N_{S},\xi ,t)]\\ & \;+\nu_{-\xi }P(N_{R},N_{S},-\xi ,t)-\nu_{\xi }P(N_{R},N_{S},\xi ,t) \end{array}$$

where $E_{R/S}^{\pm }$ are shift operators such that $E_{R/S}^{\pm }f(N_{R/S},N_{S/R},t)=f\;(N_{R/S}\pm 1,N_{S/R},t)$, and the probabilities are set to *P*(*N*_*R*_, *N*_*S*_, *ξ*, *t*) = 0 whenever *N*_*R*_ < 0 or *N*_*S*_ < 0. The last line on the right-hand side of (2.5) accounts for the random environmental switching; see black line in figure 1*b*. Since $T_{R/S}^{\pm }=0$ whenever *N*_*R*_ = 0 or *N*_*S*_ = 0, this indicates that there is extinction of *R* (*N*_*R*_ = 0) and fixation of *S* (*N*_*S*_ = *N*), or fixation of *R* (*N*_*R*_ = *N*) and extinction of *S* (*N*_*S*_ = 0). When one strain fixates and replaces the other, the population composition no longer changes while its size continues to fluctuate.^2^ The multivariate ME (2.5) can be simulated exactly using standard stochastic methods (see electronic supplementary material, section A [42]), and encodes the eco-evolutionary dynamics of the model whose main distinctive feature is the *coupling of the population size N and its composition x = N_R_/N, with DN coupled to EV*; see (2.2) and below.

## 3. Results

In this section, we analyse how the coupled demographic and environmental fluctuations shape the evolution of the fraction of *R* in cooperative AMR [31]. Our main goals are to establish the conditions under which EV and DN facilitate the eradication of *R* and reduce the size of the remaining pathogenic microbial population (see also §4).

### 3.1. Coupled environmental and demographic noise induces regimes of coexistence and dominance

The eco-evolutionary long-lived behaviour of a microbial community is chiefly captured by: (i) the expected duration of the strains coexistence (mean coexistence time, MCT, that here coincides with the unconditional mean fixation time [16,57]; see electronic supplementary material, section B.2 [42]) and (ii) by the fixation (or extinction) probability of each strain, i.e. the chance that a single strain eventually takes over the entire population (or that the strain is fully replaced by others). These properties have been extensively studied in populations of constant total size, e.g. in terms of the Moran process [14,16,44,56,57,59,69], but are far less known in communities of fluctuating size when DN is coupled to EV. To gain some insight into the behaviour of microbial co-cultures under coupled eco-evolutionary dynamics defined by equation (2.5), we compute *in silico* the *R* fixation probability, denoted by *ϕ*, and the strains coexistence probability, labelled by *P*_coex_, when the external conditions fluctuate between harsh (*K*_−_ = 120, scarce resources) and mild (*K*_+_ = 1000, abundant resources). Here, *P*_coex_ is defined as the probability that both strains still coexist for a time exceeding twice the average stationary population size *t* > 2〈*N*〉.^3^ In our simulations, we consider a wide range of the switching rate *ν* and bias *δ*, with approximately 10^3^–10^4^ realisations for each dynamic environment, and different values of the cooperation thresholds, with *N*_th_ ∼ 100. In our simulations, we respectively use *s* ∼ 0.1–0.2 and *a* ∼ 0.25–0.5 as plausible values for the resistance metabolic cost and the impact of the drug on *S* [76,77]. Our choice of *K*_±_ ensures that the dynamics is not dominated mainly by DN or EV, but by the interplay of DN and EV, and the values of the cooperation threshold *N*_th_ < *K*_−_ guarantee that the fixation of either strain or their coexistence are all scenarios arising with finite probabilities in our simulations; see below and electronic supplementary material, section A [42]. Note that, as discussed in §4.2 and electronic supplementary material, section D.3, the behaviour reported here can also be observed in big, realistic populations of *N* > 10^6^.

**Figure 2.**
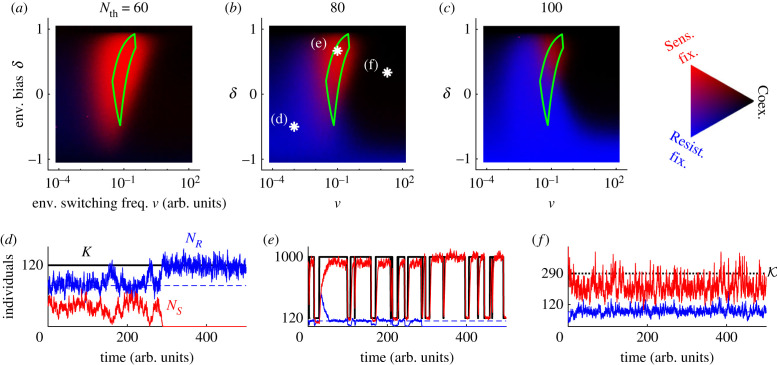
Eco-evolutionary dynamics in the phase diagram of the joint fixation and coexistence probability. (*a*–*c*) Fixation and coexistence joint probability *in silico* at a given environmental bias *δ* and mean switching frequency *ν* for *s* = 0.1, *a* = 0.25, *K*_−_ = 120 and *K*_+_ = 1000 at resistant cooperation thresholds $N_{\mathrm{th}}=60,80\;\mathrm{and}\;100$; see the discussions in §§3.3 and 4.2, and electronic supplementary material, section D.3 [42] for the behaviour at much larger populations and thresholds. Stronger blue (red) depicts a higher fixation probability of *R* (*S*). Darker colour indicates higher coexistence probability, defined as the probability to not reach any fixation before *t* = 2〈*N*〉, where we take the average total population in its stationary state. The area enclosed within the green solid line indicates the optimal regime for the eradication of *R*; see §3.4. The white asterisks in (*b*) depict the environmental statistics for each of the bottom panels. (*d*–*f*) Sample paths for the carrying capacity (*K*, black), number of *R* (*N*_*R*_, blue), number of *S* (*N*_*S*_, red) and fixed cooperation threshold *N*_th_ = 80 (dashed blue) for the environmental parameters (*ν*, *δ*) depicted by the corresponding white asterisk in (*b*). The high environmental switching frequency in (*f*) results in an effectively constant carrying capacity (K=K, dotted line); see §3.2.

Figure 2*a*–*c* shows the *in silico* *ν*–*δ* phase diagrams corresponding to the various fixation and coexistence scenarios arising for different cooperation thresholds. For small thresholds relative to EV ($N_{\mathrm{th}}≲10K_{+}/K_{-}$, see electronic supplementary material, section D.3 [42]), *S* displays a high fixation probability (red region) at intermediate *ν* and non-extreme *δ*, where *R* is most likely to be eradicated. Under high/low values of *ν* (when *δ* is not too low), the red region in figure 2*a*–*c* is surrounded by dark areas where the long-lived coexistence of the strains is most likely. When the threshold *N*_th_ is closer to *K*_−_, *R* is most likely to prevail in the blue region of figure 2*b*,*c*, where the environment is predominantly in the harsh state (*δ* < 0). As *N*_th_ increases, the blue region expands and gradually replaces the red and black areas: the fixation of *R* is likely to occur in most of the *ν*–*δ* diagram. In addition to the population make-up, the average population size is a decreasing function of *ν* at fixed *δ*, and increasing with *δ* at fixed *ν*; see figure 4*d*,*e* and §4.1, and [21–25].

In what follows, we analyse the different phases of figure 2*a*–*c*, focusing particularly on the characterisation of the red area, and also determine how *N*_*R*_ varies with the environmental parameters in the different phases. This allows us to determine *the most favourable environmental conditions for the eradication of R and for the reduction of the population of pathogenic cells*, which are issues of great biological and practical relevance.

### 3.2. Weak demographic noise promotes coexistence

To gain an intuitive understanding of the model’s eco-evolutionary dynamics, it is useful to discuss the sample paths of figures 1*b*,*c* and 2*d*–*f* in terms of the population size *N* and the *R* fraction *x* = *N*_*R*_/*N*.

It is instructive to first consider the case of very large population arising with a constant and large carrying capacity *K*(*t*) = *K*_0_ ≫ 1. In this setting, corresponding to a static environment, we ignore all forms of fluctuations and the system evolves according to the mean-field (deterministic) differential equations

3.1 $$\dot{N}=\sum_{\alpha =R,S}(T_{\alpha }^{+}-T_{\alpha }^{-})=N\left(1-\frac{N}{K_{0}}\right)$$

and

3.2 $$\dot{x}=\frac{d}{dt}\frac{N_{R}}{N}=\frac{T_{R}^{+}-T_{R}^{-}}{N}-x\frac{\dot{N}}{N}=\frac{\left(a\theta [N_{\mathrm{th}}-xN]-s)x(1-x\right)}{(1-a\theta [N_{\mathrm{th}}-xN])+(a\theta [N_{\mathrm{th}}-xN]-s)x},$$

where the dot indicates the time derivative. It is clear from equation (3.2) that the dynamics of the population composition, given by *x*, is coupled to that of its size *N*. According to the logistic equation (3.1), the population size reaches *N* = *K*_0_ on a time scale *t* ∼ 1 independently of *x*, while the population composition is characterised by a stable equilibrium *x* = *x*_th_ ≡ *N*_th_/*N* = *N*_th_/*K*_0_ reached on a time scale of *t* ∼ 1/*s* or ∼1/(*a* − *s*) from *x* > *N*_th_/*N* or <*N*_th_/*N*, respectively. When *s* < *a* ≪ 1, there is a time-scale separation, with *N* relaxing to its equilibrium much faster than *x*. We note that the coexistence equilibrium in terms of *R* and *S* is $N_{R}^{\mathrm{eq}}=N_{\mathrm{th}}$ and $N_{S}^{\mathrm{eq}}=K_{0}-N_{\mathrm{th}}$. Clearly, this suggests that *S* would unavoidably be wiped out if *N*_th_ was greater than the carrying capacity, and hence we always consider that the latter exceeds the cooperation threshold (*K* > *N*_th_).

When the population is large enough for demographic fluctuations to be negligible ($1/\sqrt{N}\to 0$) and the sole source of randomness stems from the time-fluctuating environment (random switches of the carrying capacity), the dynamics becomes a so-called piecewise deterministic Markov process (PDMP) [78]. Between each environmental switch, the dynamics is deterministic and given by equations (3.1), with *K*_0_ replaced by *K*_±_ in the environmental state *ξ* = ±1, and (3.2). Here, the PDMP is thus defined by

3.3 $$\dot{N}=N\left(1-\frac{N}{K(t)}\right)=\left\{ \begin{array}{cc} N\left(1-\frac{N}{K_{-}}\right),\; & \mathrm{if}\;\xi =-1\;\\ N\left(1-\frac{N}{K_{+}}\right),\; & \mathrm{if}\;\xi =1\; \end{array}\right.,$$

where the fluctuating carrying capacity *K*(*t*) is given by equation (2.4), coupled to (3.2). Sample paths of this PDMP are shown as solid lines in figures 1*b*,*c* and 2*d*–*f*. These realisations illustrate that *N*(*t*) tracks the switching carrying capacity *K*(*t*) independently of *x*, while *x*(*t*) evolves towards the coexistence equilibrium at the cooperation threshold *x*_th_(*t*) = *N*_th_/*N*(*t*), which changes in time as *N* varies. Hence, *x* increases when *N*_*R*_ < *N*_th_, and it decreases when *N*_*R*_ > *N*_th_. For extremely high environmental switching rate *ν* → ∞, the microbial community experiences a large number of switches, between any update of the population make-up. In this case, *N* is not able to track *K*(*t*), but experiences an effectively constant carrying capacity K=K≡1/⟨1/K(t)⟩ obtained by self-averaging the environmental noise over its stationary distribution (see [21,22,24–26]), leading to $K=2K_{+}K_{-}/[(1-\delta )K_{+}+(1+\delta )K_{-}]$. Hence, when *ν* → ∞, the community size is approximately N≈K and, provided that *δ* is not too close to −1 (K not too close to *K*_−_), long-lived coexistence of both strains is likely (with abundances *N*_*R*_ ≈ *N*_th_ and $N_{S}\approx K-N_{\mathrm{th}}$), as shown in figure 2*f*.

### 3.3. Antimicrobial resistance is robust to demographic noise in static environments

When EV causes a population bottleneck, DN about the coexistence equilibrium may cause the extinction of one strain and the fixation of the other (see figures 1*b*,*c* and 2*d*,*e*). To elucidate the fate of microbial communities under fluctuating environments, it is therefore necessary to first understand how a small community is able to fixate, or avoid extinction, in a static environment, when it is subject to a constant carrying capacity *K*_0_, with 1 ≪ *K*_0_ ∼ *K*_−_ ≪ *K*_+_. This condition ensures both fixation of one strain or long-lived coexistence are possible, i.e. demographic fluctuations, of order $O(1/{\sqrt{K}}_{0})$, matter but do not govern the dynamics.

Since the community composition tends to the coexistence equilibrium *x* → *x*_th_ (see equation (3.2)), the faster *N* dynamics reaches its steady state *N* → *K*_0_ before any fixation/extinction events occur (see equation (3.1)). Therefore, we assume a fixed *N* = *K*_0_. The evolutionary dynamics is thus modelled by the analytically tractable Moran process [14,16,21,25,58,59], where the population composition evolves stochastically by balancing each birth/death of *R* by the simultaneous death/birth of a *S*, according to the reactions

$$N_{R}+N_{S}\overset{{\tilde{T}}_{R}^{+}}{\to }\;(N_{R}+1)+(N_{S}-1)$$

and

$$N_{R}+N_{S}\overset{{\tilde{T}}_{R}^{-}}{\to }\;(N_{R}-1)+(N_{S}+1),$$

with the effective transition rates ${\tilde{T}}_{R}^{\pm }=T_{R}^{\pm }T_{S}^{\mp }/N$ obtained from (2.2) [21,25].

Due to DN, the *R* fraction fluctuates around *x*_th_ until the eventual extinction of a strain. Therefore, from the classic Moran results (electronic supplementary material, equation (S6) in [42]), we can derive a simplified, approximated expression for the *R* fixation probability by setting any initial composition directly at coexistence *x*_0_ = *x*_th_, which yields

**Figure 3.**
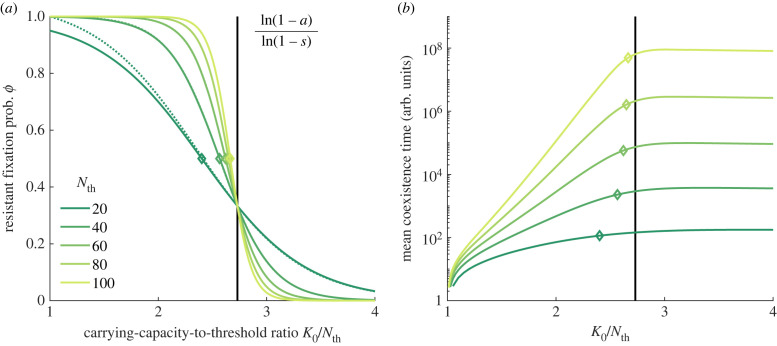
Moran theory for *R* fixation probability and mean coexistence time (MCT) in static environments. (*a*) *R* fixation probability *ϕ* in terms of the total microbial population normalised by the resistant cooperation threshold *K*_0_/*N*_th_ for five example thresholds, from *N*_th_ = 20 (dark green) to 100 (yellow green); the starting microbial composition is set at the coexistence equilibrium *x*_th_ = *N*_th_/*K*_0_; solid lines show the approximated prediction of equation (3.4); dotted lines depict the exact Moran behaviour of electronic supplementary material, equation (S6) [42], only distinguishable for the smallest threshold; open diamonds illustrate the predicted *K*_0_*/*N*_th_ that provides fixation equiprobability for each resistant cooperation threshold, see §3.3. (*b*) Mean coexistence time versus *K*_0_/*N*_th_ in log-linear scale; solid lines show the exact Moran MCT, computed from electronic supplementary material, equation (S8); legend and symbols as in (*a*).

3.4 $$\phi ≃\frac{1}{1+1/{\left(1-s\right)}^{K_{0}-K_{0}^{*}}}\;\mathrm{with}\;K_{0}^{*}\equiv N_{\mathrm{th}}\frac{\ln(1-a)}{\ln(1-s)}-\frac{\ln\left(s(1-a)/\left(a-s\right)\right)}{\ln\left(1-s\right)},$$

where we now assumed ${\left(1-a\right)}^{N_{\mathrm{th}}}\ll {\left(1-s\right)}^{N_{\mathrm{th}}}$ and ${\left(1-s\right)}^{K_{0}}\ll {\left(1-s\right)}^{N_{\mathrm{th}}}$, which is in line with our choices 0 < *s* < *a* < 1 and *N*_th_ < *K*_0_. Here, *K*_0_* is the microbial population size giving the same fixation probability 1/2 to *R* and *S*. In our examples, *s* = 0.1 and *a* = 0.25 (see §3.1), and fixation equiprobability is reached at *K*_0_* ≈ 3*N*_th_, where the *R* and *S* abundance in the long-lived coexistence equilibrium are, respectively, $N_{R}^{\mathrm{eq}}\approx K_{0}/3$ and $N_{S}^{\mathrm{eq}}\approx 2K_{0}/3$. Figure 3*a* shows the excellent agreement between the approximation (3.4) (solid lines) and the exact *R* fixation probability of the underlying Moran process of electronic supplementary material, equation (S6) (dotted lines), for different cooperation thresholds (*N*_th_ = 20–100).^4^

Equation (3.4) and figure 3*a,b* show that, in a static environment, the relative magnitude of the carrying-capacity-to-threshold ratio *K*_0_/*N*_th_ with respect to *K*_0_*/*N*_th_ ≈ ln(1 − *a*)/ln(1 − *s*) clearly determines whether *R* fixates (for smaller *K*_0_/*N*_th_), becomes extinct (larger *K*_0_/*N*_th_), or coexists with *S* for a long time (larger *K*_0_/*N*_th_ and large populations). To interpret these results, we remember that the mean field behaviour tends to *N*_*R*_ = *N*_th_ and *N*_*S*_ = *K*_0_ − *N*_th_. When *K*_0_/*N*_th_ ∼ 1, *N*_*S*_ ≈ *K*_0_ − *N*_th_ is small, and *S* is prone to extinction (*R* fixates). As the total population *K*_0_ increases (at fixed cooperation threshold *N*_th_), the equilibrium value *N*_*S*_ ≈ *K*_0_ − *N*_th_ increases, making *S* less likely to go extinct. The fixation probability of *S* thus rises, and overcomes that of the strain *R* when *K*_0_ > *K*_0_*. However, the expected time for the fixation of *S* increases exponentially with *K*_0_ and, for large enough cooperation thresholds (typically for *N*_th_ > 50), fixation takes too long and is unobservable in practice; see figure 3*b*. Note that the expected *S* fixation time (or *R* extinction time) saturates for *K*_0_ > *K*_0_* because the equilibrium value *N*_*R*_ ≈ *N*_th_ is independent of the total population *K*_0_. For all examples in figure 2*a*–*c*, we have $K_{0}=K_{+}=1000>K_{0}^{*}$ when *δ* = 1 and $K_{0}=K_{-}=120<K_{0}^{*}$ when *δ* = −1. This explains the dark areas (coexistence) in figure 2*b*,*c* where *δ* → 1, and the blue regions (*R* fixation) where *δ* → −1. In figure 2*a*, we observe dark regions (coexistence) for both *δ* = ±1 as the MCT is always larger than the coexistence threshold (*t* > 2*K*_0_). Therefore, it appears that in static environments AMR always dominates or, at least, survives for extended periods.

### 3.4. Demographic noise can eradicate antimicrobial resistance in fluctuating environments

Under low and high environmental switching rates, the community behaves as in static total populations of size *K*_±_ and K, respectively; see electronic supplementary material, sections D.1 and D.2. Richer and novel dynamical behaviour arises at intermediate switching rate (in figure 2*a*–*c*, see red areas around *ν* = 10^−2^–10^0^), when there are several environmental switches prior to fixation, and the quantities *ϕ* and *P*_coex_ cannot be simply expressed in terms of their counterparts in a population of constant effective size. This switching regime is characterised by the full interplay of the ecological and evolutionary dynamics: as shown in figures 1*b* and 2*e*, environmental switches can thus lead to transient ‘bumps’ and ‘dips’ in *N*_*R*_ (after the carrying capacity increases *K*_−_ → *K*_+_ or decreases *K*_+_ → *K*_−_, respectively). The transient *N*_*R*_ dips, together with demographic fluctuations caused by the population bottleneck (*K*_+_ → *K*_−_), can thus lead to the rapid eradication of *R* with the fixation of *S* (red areas in figure 2*a*–*c*). Each dip has a small but non-negligible probability to eradicate *R* and hence reduces the expected coexistence time. Therefore, the ingredients for this fluctuation-driven AMR eradication mechanism are: (i) intermediate environmental switching, so that the total population *N* fluctuates by tracking *K*(*t*) without lagging behind (see green lines in figure 1*b*); (ii) a slower population composition *x* coupled to the faster *N*, so that the *R* population *N*_*R*_ = *xN* experiences transient bumps and dips about its equilibrium *N*_*R*_ ≈ *N*_th_ (see blue lines in figure 1*b*,*c*); and (iii) a small number of *R* at the bottom of transient dips *N*_*R*_ ∼ 1, so that DN can drive *R* to extinction (see blue noisy line in figure 1*b*).

Here, we are interested in characterising the transient *N*_*R*_ dips as the main fluctuation-driven mechanism leading to the possible eradication of *R*. To study their properties, it is useful to consider the PDMP description of the transient *R* behaviour in large populations

3.5 $$\begin{array}{cc} & \dot{N_{R}}=T_{R}^{+}-T_{R}^{-}=\frac{\left(a-s)N_{R}(\alpha_{R}-(N_{R}/K(t))\right)}{\left(1-a)+(a-s)N_{R}/N(t\right)},\\ & \mathrm{with}\;\alpha_{R}\equiv \frac{\left(1-s)K(t)-(1-a)N(t\right)}{\left(a-s)K(t\right)}, \end{array}$$

where *K*(*t*) and *N*(*t*) are, respectively, given by equations (2.4) and (3.3), and we assume *N*_*R*_ < *N*_th_. We note that, after a switch from the mild to harsh environment (*K*_+_ → *K*_−_) in the absence of DN, *R* always survives the ensuing transient dip, and *N*_*R*_ rises towards the coexistence equilibrium *N*_*R*_ = *N*_th_; see thick solid line in figure 1*b*. However, when *K*_−_ ≪ *K*_+_ and the microbial community experiences a population bottleneck, a transient dip to a small value of *N*_*R*_ can form. When this occurs, *R* is prone to extinction caused by non-negligible demographic fluctuations (stronger when *N*_*R*_ is small).

To characterise the region of the *ν*–*δ* phase diagram where transient *N*_*R*_ dips cause eradication of *R*, we need to estimate *t*_dip_, defined as the time from the onset of the dip to when *N*_*R*_ reaches its minimal value according to equation (3.5), see figure 1*b*. To determine *t*_dip_ from (3.5), we require $\dot{N_{R}}(t_{\mathrm{dip}})=0$, which, assuming *K*_+_ ≫ *K*_−_ ≫ 1, yields *α*_*R*_ = *N*_*R*_(*t*_dip_)/*K*_−_ ≈ 0, implying *N*(*t*_dip_) ≈ *K*_−_(1 − *s*)/(1 − *a*). From the solution of equation (3.1) with the initial condition *N*(*t* = 0) ≈ *K*_+_, we find

3.6 $$t_{\mathrm{dip}}\approx \ln\left[\frac{1-s}{a-s}\left(1-\frac{K_{-}}{K_{+}}\right)\right].$$

Ignoring DN, we can thus estimate the *R* population at the bottom of the transient dip $N_{R}^{\mathrm{dip}}$, reached at *t* = *t*_dip_. This is, we find the *R* fraction at the bottom of the dip *x*(*t*_dip_) in the small *x* limit of equation (3.2) and combine it with the above *N*(*t*_dip_) to obtain (see electronic supplementary material, section D.3 [42]):

3.7 $$N_{R}^{\mathrm{dip}}=x(t_{\mathrm{dip}})N(t_{\mathrm{dip}})\approx \frac{N_{\mathrm{th}}K_{-}}{K_{+}}\frac{1-s}{1-a}{\left(\frac{1-s}{a-s}\right)}^{\left(a-s)/(1-a\right)}≳\frac{N_{\mathrm{th}}K_{-}}{K_{+}},$$

where we assumed that *R* started from *N*_*R*_(*t* = 0) = *N*_th_. Demographic fluctuations at the bottom of a dip are of the order $\sqrt{N_{R}^{\mathrm{dip}}}$. For DN to possibly drive *R* to extinction, and the fluctuation-driven eradication scenario to hold, it is necessary that $\sqrt{N_{R}^{\mathrm{dip}}}∼N_{R}^{\mathrm{dip}}$, which requires $N_{R}^{\mathrm{dip}}=O(1)$, i.e. $N_{R}^{\mathrm{dip}}∼10$ or lower. This condition is certainly satisfied when *K*_−_ and *N*_th_ are of comparable size (with *K*_−_ > *N*_th_), and each of order $\sqrt{K_{+}}$, which can also hold for realistically large populations of *N* > 10^6^, see §4.2 and electronic supplementary material, section D.3 [42].

Under these sufficient requirements, the optimal environmental conditions to rapidly eradicate *R* in large but fluctuating populations can be estimated from equations (3.1), (3.2), (3.5) and (3.6). First, in the mild environment (*K* = *K*_+_), *R* needs to be able to evolve to the coexistence equilibrium *N*_*R*_ = *N*_th_, requiring a longer average duration of the mild environment $\nu_{+}^{-1}$ when compared with the evolutionary time scale *s*^−1^, i.e. $\nu_{+}^{-1}≳s^{-1}$. Second, after the switch from mild to harsh environment (*K*_+_ → *K*_−_), *R* needs to reach the bottom of the transient dip and experience demographic fluctuations, which imposes an average duration of the harsh environment $\nu_{-}^{-1}$ longer than the average time to reach the bottom of the dip *t*_dip_, that is, $\nu_{-}^{-1}≳t_{\mathrm{dip}}$. Third, if *R* survives the dip, the environment should go back to the mild *ξ* = 1 state to rule out the extinction of *S* when the environment stays in the harsh state *ξ* = −1. For this, we require the harsh environment to be short, while ensuring that the dip is not interrupted by a switch; see figure 1*b*,*c*. This enforces $\nu_{-}^{-1}≲2\ln\left(K_{+}/K_{-}\right){\left(a-s\right)}^{-1}$, where the right-hand side, derived from the small *x* limit of equation (3.2), is twice the expected time to reach the equilibrium in the harsh state *N*_*R*_ = *N*_th_ and *N*_*S*_ = *K*_−_ − *N*_th_. As a fourth condition, we demand that this cycle should be as fast as possible to maximise the number of transient dips (while still allowing the population to evolve back to *N*_*R*_ = *N*_th_ after a bump), yielding $\nu_{+}^{-1}≲2\ln\left(K_{+}/K_{-}\right)s^{-1}$, which, similarly as in the previous condition, is twice the average time needed to return to equilibrium in the mild state. Using the environmental parameters *ν* and *δ*, the above leads to

3.8 $$\begin{array}{cc} & \frac{s}{2\ln\left(K_{+}/K_{-}\right)}≲\nu_{\mathrm{opt}}(1-\delta_{\mathrm{opt}})≲s\\ \mathrm{and}\; & \frac{a-s}{2\ln\left(K_{+}/K_{-}\right)}≲\nu_{\mathrm{opt}}(1+\delta_{\mathrm{opt}})≲\frac{1}{t_{\mathrm{dip}}}. \end{array}$$

The green contour lines in figure 2*a*–*c* enclose the predicted optimal region for the fast eradication of *R* under *s* = 0.1, *a* = 0.25, *K*_−_ = 120 and *K*_−_ = 1000, and fall in the red areas observed *in silico*. The borders of these regions depend on *N*_th_. This stems from the dependence of *ϕ* and MCT on *N*_th_ (figure 3*b*) and the criterion for long-lived coexistence (*t* > 2〈*N*〉). The conservative prediction (3.8) ignores any dependence on *N*_th_.

In summary, DN can eradicate AMR in fluctuating environments when the population make-up *x* evolves on a much slower time scale than the population size *N*, which requires relatively small values of *s* and *a*. Moreover, the variability in the carrying capacity *K*_+_/*K*_−_ needs to be of the order of the cooperation threshold *N*_th_ or larger; the threshold has to fall below the lowest value of the carrying capacity *K*_−_; and the switching rate *ν* has to be of order *s* and hence comparable to the rate of relaxation of the population composition. Note that all conditions above can be met in biologically relevant systems of any size; see §4.2 and electronic supplementary material, section D.3 [42].

## 4. Discussion

The results of the previous section characterise the long-term microbial population make-up under random switches between mild and harsh environmental conditions (high and low carrying capacity, *K* = *K*_+_ and *K*_−_, respectively), for a broad range of the exogenous parameters (mean switching frequency *ν* and switching bias *δ*). Another important aspect of the time evolution of microbial population concerns the non-trivial impact of the EV on the fraction and abundance of drug-resistant (*R*) and drug-sensitive (*S*) microbes in the different regimes, and especially in their phase of coexistence. It is also important to review to what extent our modelling assumptions are amenable to experimental probes.

### 4.1. Impact of environmental variability on the strains fraction and abundance

**Figure 4.**
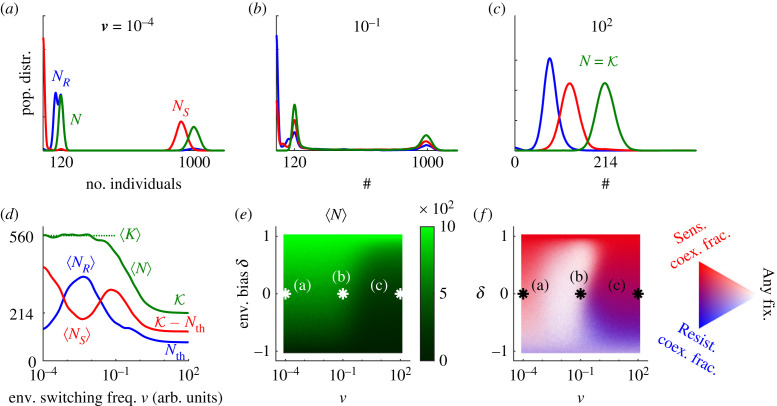
Total population, strain abundance and coexistence composition in fluctuating environments. (*a*–*c*) *In silico* probability distributions of the total population (*N*, green), number of *R* (*N*_*R*_, blue) and number of *S* (*N*_*S*_, red), with parameters *s* = 0.1, *a* = 0.25, *K*_−_ = 120, *K*_+_ = 1000 and *N*_th_ = 80, under no environmental bias (*δ* = 0) and for mean switching rates in slow *ν* = 10^−4^ (*a*), intermediate *ν* = 10^−1^ (*b*), and fast *ν* = 10^2^ (*c*) conditions. Histograms are smoothed by a Gaussian filter of width *σ* = 10 in cell number. (*d*) Average overall population (number of individuals on the vertical axis) and strain abundances under no bias, i.e. *δ* = 0, as a function of switching rate *ν*; colours as in (*a*–*c*). Lines are smoothed by a log-scale Gaussian filter of width *σ* = 10, i.e. one frequency decade. (*e*) Average overall population size in dynamic environments. (*f*) Coexistence composition and fixation probability (of any strain) in dynamic environments. Stronger blue (red) depicts a higher coexistence fraction of *R* (*S*). Lighter colour indicates lower coexistence probability, defined as the probability for no fixation event to occur before *t* = 2〈*N*〉. The white and black asterisks in (*e*,*f*) depict the environmental statistics for each of the top panels. All panels are computed at quasi-stationarity reached after a time *t* = 2〈*K*〉, ensuring that *N* reaches its (quasi-)stationary state, where 〈*N*〉 ≤ 〈*K*〉; see text.

It was recently shown that in the fluctuating environment considered here, the average size of the microbial community 〈*N*〉 is a decreasing function of the random switching rate *ν* (with *δ* kept fixed), that 〈*N*〉 decreases with lower *δ* (keeping *ν* fixed), and that 〈*N*〉 →*K*_±_ as *δ* → ±1 [21–23,25]; see figure 4*d*,*e*. As a consequence, in the blue and red areas of the phase diagrams of figure 2, where only one strain survives (figure 2*a*–*c*), the surviving pathogenic population can be reduced by increasing the environmental switching frequency *ν* and/or the time spent in harsh state (by enforcing *δ* → −1). Moreover, since the *R* fraction *x* is directly coupled to *N* through the cooperation threshold *N*_th_ (see equation (3.2)), EV non-trivially shapes the *R* fraction in the coexistence regime (coloured areas in figure 4*f*).

Under low switching frequency relative to the rate of evolutionary dynamics (see ν≪ resistance extra metabolic cost *s* ∼ 10^−1^ in all figures), *R* cells starting in the mild environment (*K*_+_) are able to reach the coexistence equilibrium *N*_*R*_ = *N*_th_ before experiencing a switch; see figure 1*c*. However, if the starting environment is harsh (*K*_−_), DN can rapidly eradicate *S* and destroy coexistence (figure 2*b*,*c*). The distributions of *N*_*R*_, *N*_*S*_ and *N* in the regime *ν* → 0 are thus approximately bimodal because they combine both mild and harsh (effectively constant) environments; with *N*_*R*_ ≈ *N*_th_, *N*_*S*_ ≈ *K*_+_ −*N*_th_ and *N* ≈ *K*_+_ for the former; and *N*_*R*_ ≈ *K*_−_, *N*_*S*_ ≈ 0 and *N* ≈ *K*_−_ for the latter; see figure 4*a*. As the switching rate is increased to ν≲s, fixation dominates, and the *N*_*R*_ and *N*_*S*_ bimodal distributions become approximately trimodal, i.e. *N*_*R*/*S*_ ≈ 0, *K*_−_ or *K*_+_; see figure 4*b*. The relative weight of the peaks at *N*_*R*/*S*_ ≈ 0 is set by *S*/*R* fixation probability, which is modulated by the environmental bias *δ*, see electronic supplementary material, equation (S9) [42]. The total population distribution is still bimodal about *N* = *K*_±_ since its relaxation dynamics (of time scale ∼1; see equation (3.1)) is faster than the evolutionary time scale of approximately *s*^−1^. Finally, when *ν* is increased further (*ν* ≫ *s*), we enter the coexistence regime characterised by an effective carrying capacity K=K [21–26], and all distributions become unimodal about the coexistence equilibrium *N*_*R*_ ≈ *N*_th_, $N_{S}\approx K-N_{\mathrm{th}}$ and N≈K; see figure 4*c*.

As a consequence, if *R* is eradicated, imposing high EV (*ν* ≫ *s*) and harsh conditions *δ* → −1 would considerably reduce the abundance of the surviving community of pathogenic *S* cells; see figure 4*d*, green solid line, and figure 4*e*. However, if *R* survives, imposing *ν* ≫ 1 and *δ* < 0 would not only decrease the abundance of both strains but it would also increase the *R* fraction, and risk further AMR spreading, see figure 4*f* (magenta/bluish areas).

### 4.2. Review of the modelling assumptions

Since we study an idealised microbial model, it is important to review our modelling assumptions in light of realistic laboratory experimental conditions. A key assumption to consider is the effectively sharp cooperation threshold *N*_th_, which is based on a number of experimental observations of microbial cooperation; see [34–36,52]. Accordingly, we have assumed that EV changes chemical concentrations (e.g. nutrient density) while the volume of the microbial ecosystem is kept constant [52]. The cooperation threshold is then fixed at a constant number of *R* microbes *N*_*R*_ = *N*_th_ because, at constant volume, the resistance enzyme concentration is proportional to the number of public good producers *R*. This crucial ingredient fixes the stable number of *R* at *N*_th_ across fluctuating environments, and is responsible for the transient dips which are at the origin of the novel eco-evolutionary mechanism for the eradication of AMR reported here. The complementary scenario, where the cooperation threshold is set by a fixed *R* fraction *x*_th_ is also relevant (for a different set of microbial ecosystems), and is a topic for future research. Furthermore, in some microbial cases, *R* could regulate the production of resistance enzyme by quorum sensing [79], but its impact on cooperative AMR remains an open problem. We also note that some resistance mechanisms can show anti-cooperative behaviour, such as efflux-pumps, which could result in an enhanced exposure of sensitive cells to the drug [80,81]. In the case of non-shared resistance mechanisms, our model reduces to the eco-evolutionary processes studied in [21–23,25]. Further analytical results for the non-shared resistance models are discussed in [82], as well as in [83–85] in the case of a static environment.

A second assumption to review concerns the simulation results obtained here, for systems with *K*_±_ ∼ 10^2^−10^3^ and *N*_th_ ∼ 100, that we are able to computationally probe (see electronic supplementary material, section A [42]) but that correspond to populations of relatively small size. In electronic supplementary material, section D.3, we provide a detailed discussion on how the rich microbial behaviour and novel eco-evolutionary AMR eradication mechanism reported here can be translated to larger, more realistic, microbial communities of size of order $N≳10^{6}$ [52] to $N≳10^{8}$ [31,86–89], or higher. In our discussion, we argue that, as long as $N_{\mathrm{th}}K_{-}/K_{+}≲10$ and $0<s<a≲10^{-1}$–10^−^^2^, regardless of the magnitude of *K*_±_ or *N*_th_, the transient dips studied here will drag *R* close to extinction, where demographic fluctuations are instrumental for the likely and rapid eradication of AMR. Note that, for very fast/slow fluctuating environments, where transient dips are hindered (see §3.4), *R* and *S* populations will always coexist unless $K_{-}-N_{\mathrm{th}}≲10$.

A specificity of our study is its focus on biostatic antimicrobial drugs. However, since most antimicrobials gradually change from acting as biostatic to biocidal as their concentration in the medium grows [53–55], our approach is consistent with a low antimicrobial concentration scenario. Conveniently, the combined biostatic effect of the drug and the normalisation of strain fitness in equation (2.2) [14,16] decouples the total population *N* from its composition *x*. If any of the above conditions would not hold, *N* would then directly depend on *x*, a case already studied for a simpler model in [21,25]. It is worth noting that we have confirmed that the main findings reported here are robust, as they do not depend crucially on the detailed choice of the transition rates in equation (2.2), and in particular they are found to be essentially independent of the normalisation by the average fitness; see §§2.1 and 3.3. We also note that the values used in our examples for the extra metabolic cost to generate the resistance enzyme (s∼10% to 25%), and for the impact of the antimicrobial drug on *S* growth (a∼25% to 50%), while only indicative, are plausible figures [76,77].

For the sake of simplicity, we have focused on modelling EV through binary switches of the carrying capacity. These switches capture sudden changes in the available resources (as in feast and famine cycles [1–3,60]) that can also occur in the presence of antimicrobial drugs, e.g. in polluted environments or during drug treatment. In the context of evolutionary processes, environments that fluctuate via random switches are commonly modelled in terms of dichotomous Markov noise (DMN), also known as telegraph noise [22–26,32,46–48,61,62]. Moreover, binary switching is the standard way to implement EV in laboratory-controlled experiments, where the concentration of nutrients can be regulated in a chemostat set-up [63–66]. Although laboratory experiments are often carried out with periodically switching environments (e.g. [63,64]), and natural environmental conditions often vary continuously in time and magnitude (e.g. [66]), the relationship between DMN and other commonly used forms of EV has already been extensively studied [24,46,47]. Therefore, our choice of modelling EV with DMN is natural, convenient and non-limiting: it allows us to make mathematical progress while keeping the theoretical modelling close to laboratory experimental conditions. The literature suggests that the essence of our findings are expected to hold for general fluctuating environments with a time-varying carrying capacity, but the extent to which other and more complex forms of EV than binary random switching may alter our results for microbial communities exhibiting cooperative AMR remains a problem to be studied.

Finally, we note that the novel eco-evolutionary mechanisms reported in this study to eradicate cooperative AMR, and to reduce the total pathogenic microbial community, or minimise the coexistence fraction of *R*, all take place at a biologically and clinically relevant range of environmental switching rates. Indeed, although our theoretical study does not set a specific time scale of microbial growth, a plausible rough estimate for a single replication cycle of a microbe could be of the order of approximately 1 h. The novel AMR eradication mechanism at *ν* ∼ *s* then comes into play when a single environmental phase lasts, on average, *s*^−1^ ∼ 10 h. This could be consistent with the periodic administration of a treatment that enforces microbial population bottlenecks, and is a feasible time scale for laboratory experiments. Our idealised model, however, assumes a homeostatic influx of antimicrobial drug in all environments. Thus, an interesting approach for future work would involve the joint application of antimicrobial drug and population bottlenecks (in the harsh environment), with no drug administered in the mild environmental state.

## 5. Conclusion

Understanding how EV affects the demographic and ecological evolution of microbes is central to tackle the threat of AMR, an issue of pressing societal concern [33]. Central questions in studying AMR involve how the fraction of resistant microbes changes in time, and by what mechanisms these can possibly be eradicated.

It is well established that AMR is an emergent property of microbial communities, shaped by complex interactions. In particular, certain resistant cells able to inactivate antimicrobials can, under certain conditions, protect the entire microbial community. This mechanism can hence be viewed as an AMR cooperative behaviour. Moreover, microbial populations are subject to changing conditions. For instance, the size of a microbial population can vary greatly with the variation of the nutrients or toxins, and can e.g. experience bottlenecks. As a result of evolving in volatile environments, microbial communities are prone to be shaped by fluctuations. In general, these stem from EV (exogenous noise) and, chiefly in small populations, from DN. The underlying eco-evolutionary dynamics, characterised by the coupling of DN and EV, is ubiquitous in microbial ecosystems and plays a key role to understand the AMR evolution, but is still rather poorly understood.

In this work, we have studied an idealised model of cooperative AMR where a well-mixed, microbial population consisting of sensitive and resistant cells is treated with an antimicrobial (biostatic) drug, hindering microbial growth, in a fluctuating environment. The latter is modelled by a binary switching carrying capacity that fluctuates between two values corresponding to mild and harsh conditions (high/low values, respectively). Based on a body of experimental work [34–39], we assume that resistant cells produce, at a metabolic cost, an enzyme that inactivates the antimicrobial drug. Importantly, the abundance of resistant microbes is thus a proxy for the concentration of the drug-inactivating enzyme, which above a certain abundance threshold, becomes a public good by providing drug protection, at no metabolic cost, to the sensitive strain. Above the cooperative threshold, the latter hence have a fitness advantage over the resistant strain, whereas, below the threshold, the drug is responsible for a reduced fitness of the sensitive cells. In this setting, the evolution of AMR can be viewed as a public good problem in a varying environment, whose outcome is shaped by the coupling of environmental and demographic fluctuations.

We have identified three regimes characterising the eco-evolutionary dynamics of the model, associated with the fixation of the resistant or sensitive microbes, or with the long-lived coexistence of both strains. Our analysis shows that, while AMR generally survives, and often prevails, in static environments, a very different scenario can emerge under EV. In fact, we demonstrate that fluctuations between mild and harsh conditions, coupled to DN, can lead to ‘transient dips’ in the abundance of resistant microbes, which can then be driven to extinction by demographic fluctuations. Here, we determine that this *fluctuation-driven AMR eradication mechanism* occurs when the rate of environmental change is comparable to that of the relaxation of the evolutionary dynamics (*ν* ∼ *s*). By computational means, we show that this fluctuation-driven mechanism speeds up the eradication of resistant cells, and argue that it holds also for large microbial communities, comparable to those used in laboratory experiments (*N* > 10^6^). We have also studied how EV non-trivially affects the strain abundance in the various regimes of the model, and in particular have determined the complex long-lived distribution of the fraction of resistant cells when both strains coexist and the environment fluctuates.

In conclusion, we have shown the existence of a biophysically plausible novel mechanism, driven by the coupling of EV and DN, to eradicate resistant microbes, and have demonstrated how EV shapes the long-lived microbial population in the possible scenarios of strains coexistence or fixation. Our work thus paves the way for numerous possible applications, for instance, in microbial experiments with controlled environmental fluctuations (it is currently possible to track even individual microbes; e.g. [90,91]), which might shed light on new possible treatments against AMR in real-world clinical infections.

## Data Availability

Simulation data and codes for all figures are electronically available from the University of Leeds Data Repository: https://doi.org/10.5518/1360. Technical and computational details are provided in electronic supplementary material [42].

## Endnotes

1 Here, for simplicity, we focus on biostatic drugs that reduce growth rate of sensitive cells *S*. Biocidal drugs would increase the death rate of *S*. In fact, our choice is not particularly limiting since the effect of a same drug can be either biostatic or biocidal, depending on the concentration of cells, and antimicrobial [53–55].

2 The model will finally settle in the absorbing state *N*_*R*_ = *N*_*S*_ = 0, which corresponds to the eventual extinction of the entire population. This occurs after a time that grows exponentially with the system size and that is unobservable when, as here, *K*(*t*) ≫ 1 [21,23,25,68]. This phenomenon, irrelevant for our purposes, is not considered here.

3 The rationale is that the MCT in two-strategy evolutionary games scales linearly with 〈*N*〉 in neutral regimes, exponentially with 〈*N*〉 in coexistence regimes and sublinearly with 〈*N*〉 in regimes where a strain dominates [57,70–73]; see also [74,75]. Therefore, *t* > 2〈*N*〉 is a conservative proxy of coexistence, since it allows us to distinguish between regimes where one of the strains dominates and fixates in a time *t* ≤ 2〈*N*〉 from a phase of long-lived coexistence (prior to the eventual fixation of one strain, after a time practically unobservable when 〈*N*〉 ≫ 1).

4 We note that, in the full model simulations at static environments, the total population *N* is not fixed but fluctuates about *K*_0_. In electronic supplementary material, section C [42], we discuss the minor quantitative impact of these *N* fluctuations on the fixation probability and MCT, see electronic supplementary material, figure S1.
